# The descriptive epidemiology of coronavirus disease 2019 during the epidemic period in Lu'an, China: achieving limited community transmission using proactive response strategies

**DOI:** 10.1017/S0950268820001478

**Published:** 2020-07-02

**Authors:** Wei Qin, Jie Sun, Pengpeng Xu, Tianqi Gong, Xiude Li, Lei Liu, Jieying Hu, Yao Wang, Shaoyu Xie, Kaichun Li, Hongwei Chang, Yong Lyu

**Affiliations:** Lu'an Municipal Center for Disease Control and Prevention, Lu'an, Anhui, China

**Keywords:** Asymptomatic carrier, cluster, coronavirus disease 2019, outbreak

## Abstract

Hubei province in China has had the most confirmed coronavirus disease 2019 (COVID-19) cases and has reported sustained transmission of the disease. Although Lu'an city is adjacent to Hubei province, its community transmission was blocked at the early stage, and the impact of the epidemic was limited. Therefore, we summarised the overall characteristics of the entire epidemic course in Lu'an to help cities with a few imported cases better contain the epidemic. A total of 69 confirmed COVID-19 cases and 11 asymptomatic carriers were identified in Lu'an during the epidemic from 12 January to 21 February 2020. Fifty-two (65.0%) cases were male, and the median age was 40 years. On admission, 56.5% of cases had a fever as the initial symptom, and pneumonia was present in 89.9% of cases. The mean serial interval and the mean duration of hospitalisation were 6.5 days (95% CI: 4.8–8.2) and 18.2 days (95% CI: 16.8–19.5), respectively. A total of 16 clusters involving 60 cases (17 first-generation cases and 43 secondary cases) were reported during the epidemic. We observed that only 18.9% (7/37) index cases resulted in community transmission during the epidemic in Lu'an, indicating that the scale of the epidemic was limited to a low level in Lu'an city. An asymptomatic carrier caused the largest cluster, involving 13 cases. Spread of COVID-19 by asymptomatic carriers represents an enormous challenge for countries responding to the pandemic.

## Introduction

Since the outbreak of coronavirus disease 2019 (COVID-19) was reported in Wuhan, China, the disease has spread from Wuhan to throughout China as well as to other countries and has become a worldwide pandemic [1, 2]. The number of confirmed cases of COVID-19 is updated daily and is expected to increase further in countries outside of China [3, 4], some of which have seen continued community transmission [5]. Lu'an city is in central China, next to Hubei province, where the most confirmed COVID-19 cases and sustained transmission have been reported. However, we were able to block transmission in Lu'an, thus preventing further spread caused by the case imported from Hubei and limiting the scale of the epidemic. We summarised the overall characteristics of the course of the entire epidemic in Lu'an, with a hope that this valuable information will help cities with few imported cases to effectively contain the COVID-19 epidemic.

## Materials and methods

### Setting

**Fig. 1.**
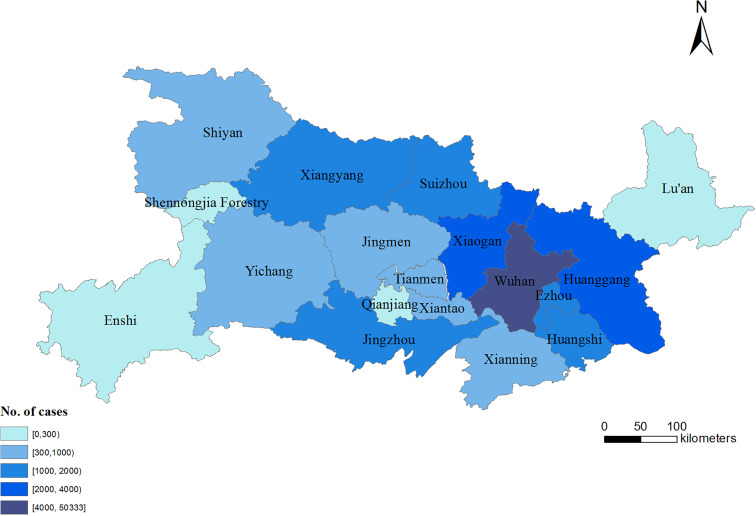
Distribution of COVID-19 cases in Hubei province and Lu'an city (as of 9 May 2020).

As shown in Figure 1, Lu'an city is located in the central region of China, adjacent to Hubei province. It is the largest city in Anhui province, with an area of 15 451 square kilometers and about 5.88 million permanent residents. The distance between Lu'an and Wuhan, the capital of Hubei province, is about 300 kilometers. Transportation between the two cities is very convenient, taking about 1.5 h by high-speed train. There are at least 30 000 Lu'anese working in Hubei, and 20 000 people typically return to Lu'an during the Spring Festival.

### Definitions

We defined cases based on the Diagnosis and Treatment Protocol of COVID-19 issued by the Chinese National Health Commission. A suspected case was defined as any person with any relevant epidemiological history plus any two clinical manifestations (fever and/or respiratory symptoms, imaging evidence of pneumonia and normal/decreased white-cell count or decreased lymphocyte count), or all three aforementioned clinical manifestations if there was no clear epidemiological history. A confirmed case was defined as a suspected case with respiratory or blood specimens that tested positive for new coronavirus nucleic acid by real-time fluorescent polymerase chain reaction (RT-PCR). We defined an asymptomatic carrier as any person with a positive result for new coronavirus nucleic acid by fluorescent RT-PCR without any clinical symptoms. A cluster of COVID-19 was defined as two or more confirmed cases or asymptomatic carriers that were found within 14 days in a small area (e.g. a family, work unit or building site) with a clear epidemiological link among those cases. Community transmission was identified if one of the following situations was met: (1) the source of infection for two or more confirmed cases or asymptomatic carriers living in the same community or village was unclear, (2) the number of clusters (⩾3) in the same community or village was large, showing a continuous trend of transmission or (3) the scale of a cluster was large, and the cluster-associated cases were distributed in at least two households, communities or villages. We defined a sporadic case as having no epidemiological link to any known confirmed case or asymptomatic carrier that did not result in a cluster later. A first-generation case was defined as the index case for each cluster.

### Data source

All suspected cases, confirmed cases and asymptomatic carriers were documented by clinical doctors in the Chinese Information System for Disease Control and Prevention (CISDCP), an internet-based, passive infectious disease surveillance system in China. Since the beginning of the COVID-19 outbreak, the CISDCP has directed local Center for Disease Control and Prevention (CDC) offices to launch comprehensive field investigations, conducted by CDC's field epidemiology investigation team (FEIT). The FEITs completed an epidemiological investigative report on each case that was discussed among team members. If some details of the report were unclear or more information was needed, an additional investigation was launched. All the data for this study were generated and collected from those field reports, including demographic and clinical information, cluster and community information, source of infection, epidemiological history, distribution through the community and characteristics of the cluster.

## Results

### Overview of epidemiology and clinical characteristics

**Fig. 2.**
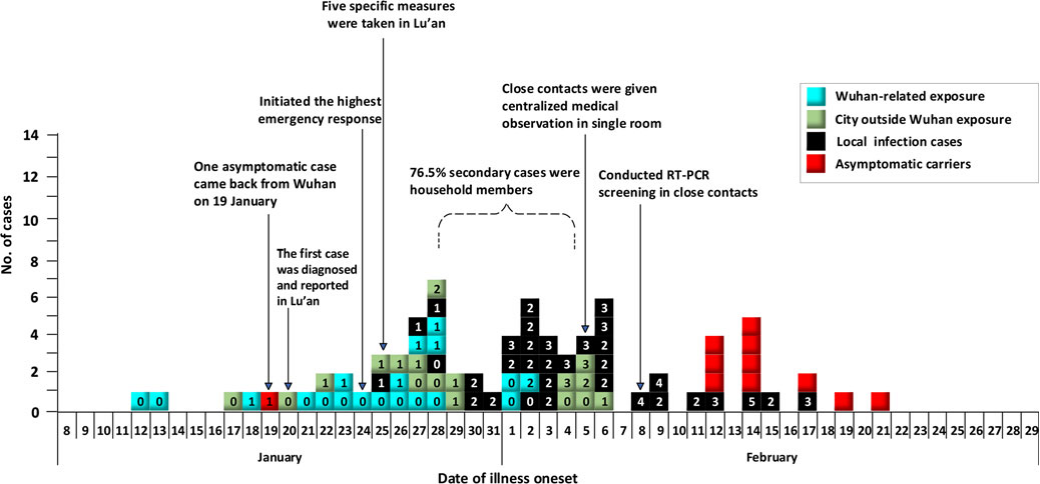
Epidemic curve of the COVID-19 epidemic in Lu'an Anhui, China, 2020.

Between 12 January and 21 February 2020, a total of 69 (86.3%) confirmed COVID-19 cases and 11 (13.7%) asymptomatic carriers were identified in Lu'an. The source of infection for 75 cases (93.8%) was identified by field investigation and traceback. As shown in Figure 2, the epidemic lasted almost 40 days, and the onset of illness peaked from 27 January to 6 February. All hospitalised cases were discharged before 4 March, with no deaths reported. Of the 80 cases, 52 (65.0%) were male and 28 (35.0%) were female. The median age was 40 years (range: 10 months to 77 years), with most cases (81.3%) aged between 20 and 60 years. Of the 69 confirmed cases, 39 (56.5%) had a fever as the initial symptom, and pneumonia was present in 89.9% of the patients on admission. Other symptoms such as cough (37.3%), weakness (14.5%), chills (8.7%) and headache (7.2%) are summarised in Table 1.

**Table 1.** Demographic and clinical characteristics among COVID-19 cases in Lu'an, Anhui, China, 2020

Characteristics	Number of cases (*N* = 80)	Percentage
Gender		
Male	52	65.0
Female	28	35.0
Age, median (range) − years	40 (0.8–77)	
Initial symptoms or signs^a^		
Pneumonia	62	89.9
Fever	39	56.5
Cough	26	37.7
Generalised weakness	10	14.5
Chills	6	8.7
Headache	5	7.2
Sore throat	3	4.3
Myalgia	2	2.9
Duration of hospitalisation, mean (95% CI) − days^b^	18.2 (16.8–19.5)	
Time to recovery, mean (95% CI) − days^b^	21.8 (20.3–23.3)	
Number of cluster-associated cases	60	75.0
Number of cases in each cluster, median (range)	3 (2–13)	
Serial interval, mean (95% CI) − days^c^	6.5 (4.8–8.2)	

a We used the confirmed COVID-19 case (*n* = 69) as the denominator.

b Since all asymptomatic carriers were only isolated in the hospital and received treatment, we used 69 confirmed cases to calculate the duration of hospitalisation and the time to recovery.

c Based on 32 pairs of cases with a clear infector and infectee relationship in 16 clusters.

For all confirmed cases, we estimated the mean time to recovery was 21.8 days (95% CI: 20.3–23.3), and the mean duration of hospitalisation was 18.2 days (95% CI: 16.8–19.5). We conducted RT-PCR screening in close contacts from 8 February to 22 February. In total, 3596 respiratory or blood specimens were collected and tested, 15 (0.4%) of which were positive. We observed that four out of 15 initially asymptomatic cases developed fever and/or respiratory symptoms within 1–5 days after the screening.

### Cluster description

As shown in Figure 2 and Table 1, of the 80 COVID-19 cases reported in Lu'an, 60 (75.0%) were cluster-associated cases, and 20 (25.0%) were sporadic cases. In total, we identified 16 clusters that were composed of 60 cases during the epidemic from 12 January to 21 February 2020. Each cluster involved a minimum of two cases and a maximum of 13 cases. We regarded the index case for each cluster as a first-generation case. We found 17 first-generation cases and 43 secondary cases. Among the 43 secondary cases, 10 asymptomatic carriers screened from close contacts were included, and all were Lu'an residents. We found the time interval between two cases (husband and wife) in one cluster was only one day, so we considered both to be first-generation cases in that cluster. Based on 32 pairs of cases with a clear infector and infectee relationship in 16 clusters, we estimated that the serial interval distribution had a mean of 6.5 days (95% CI: 4.8–8.2).

The generations and exposure of the cases are shown in Figure 2. Field investigation results showed that 20 cases returned to Lu'an from Wuhan before onset, indicating that they might have been infected in Wuhan or related to Wuhan. As shown in Figure 2, 17 cases were due to exposures outside of Wuhan during the epidemic. All patients worked in Wuhan but had returned to Lu'an during the Spring Festival. We found that 13 cases had contact with patients from Wuhan in the city where they had been working before symptom onset, suggesting they may have been infected by this exposure. The other four cases were secondary cases of infection originating in Lu'an. Among 20 Wuhan-related cases, only 25.0% (5/20) of cases generated secondary cases, and 75.0% (15/20) of Wuhan-related cases did not. Among 43 secondary cases, 38 (88.4%) were residents with no travel or residence history outside Lu'an. We found that secondary cases mainly occurred in household members, accounting for 69.8% (30/43). The largest cluster involving 13 cases was caused by an asymptomatic carrier who came back from Wuhan on 19 January 2020. We found up to at least fifth-generation cases in this cluster, and the exposure mode of the secondary cases included those who lived together, attended parties or ate together and those who shared entertainment activities.

### Transmission at the community level

In this study, we regarded communities where the sporadic cases (20 cases) and the index cases of each cluster (17 cases) were found as potential community transmission settings. We found 37 cases were distributed across 36 communities. Inevitably, these so-called initial cases may infect more and more people in other communities without effective control measures. However, we observed that only 18.9% (7/37) of these cases seeded the subsequent community transmission. In addition, the number of communities with confirmed cases or asymptomatic carriers increased from 36 to 47 during the epidemic period in Lu'an. The median for cluster-associated cases in each community was two cases (range: 1–7). Of the seven initial cases that caused community transmission, only one was reported having resided in or having visited Wuhan. He was an asymptomatic carrier, so he did not take intense quarantine and social distancing measures before he was identified. The other six initial cases that caused community transmission were characterised as exposure to cities outside of Wuhan (five cases) or as a local resident with an unknown source of infection (one case). The results showed that community transmission was more prone to occur if the epidemiological history of the initial case was unclear.

## Discussion

Although COVID-19 is highly contagious and has spread rapidly from Wuhan throughout China [6], we demonstrated the ability to block transmission and prevented further spread in Lu'an, limiting the impact and the scale of the epidemic. As a city with 5.88 million permanent residents [7], the crude COVID-19 incidence of only 1.4 per 100 000 population during the epidemic period was seemingly acceptable. Here, we summarised the characteristics of the course of the entire epidemic in Lu'an to inform the response for regions with imported cases at the early stage of spread.

Mathematical models have estimated that the reproductive number of COVID-19 was about 2.3 [8, 9], which means 20 Wuhan-related cases may have caused at least 46 secondary cases during one maximum incubation period in Lu'an. However, we found only five (25.0%) Wuhan-related, confirmed secondary cases at the beginning of the COVID-19 epidemic in Lu'an, suggesting the implementation of containment measures had worked effectively. Before the first confirmed COVID-19 case was reported in Lu'an, the city had been facing the most urgent threat from COVID-19 due to its proximity to Hubei province and the mass movement of 20 000 people from Hubei to Lu'an during the Spring Festival. However, the infection status of those people was unpredictable. Therefore, five specific measures were taken by the local government for people who came back from Wuhan, which included distributing a letter of sympathy, a card for reporting one's condition, a card with COVID-19 prevention information, a thermometer for temperature measurement and a bottle of cleaning product for daily disinfection. Additionally, before and at the early stage of the epidemic, knowledge about disease prevention (such as self-isolation and social distancing) had been popularised to improve public health literacy and skills. Our results showed that most community transmissions were not caused by Wuhan-related cases, which indicated that a series of public health interventions were associated with improved control of the COVID-19 outbreak in Lu'an. Most importantly, the non-pharmaceutical outbreak containment strategies in China, including public health interventions, appear to have been effective [10–12]. Recent studies showed that the transmission window in the community was shortened when infectious individuals were isolated more quickly [13]. Fortunately, at the early stages of the epidemic and even as it progressed, strict containment measures such as movement and traffic restrictions, self-isolation, centralised medical observation, social distancing and closure of public places were taken, which might have contributed to interrupting local COVID-19 transmission in Lu'an.

We observed that only seven (18.9%) index cases resulted in community transmission during the epidemic in Lu'an. Interestingly, community transmission was not caused by those Wuhan-related cases. In contrast, it was caused by cases of infections in cities outside of Wuhan in residents with unknown sources of infection or by those who were asymptomatic carriers. Since the epidemiological history of those cases was not obvious, the diagnosis of COVID-19 and implementation of related control measures such as isolation were delayed. Therefore, parties and other social activities continued as usual before the cases had been diagnosed, which may have increased the risk of community transmission. We also found that the largest cluster outbreak was caused by an asymptomatic carrier who came back from Wuhan, which further supports the human-to-human transmission of COVID-19 from asymptomatic carriers [14, 15]. At first, the patient had noted the risk of infection at Wuhan, so he had a physical examination on 23 January (this third day back from Wuhan) with normal findings. Frustratingly, this favourable outcome might have promoted reduced patient vigilance in implementing infection control measures. Had his infection been immediately identified and had he subsequently isolated, the number of COVID-19 cases in Lu'an might have been decreased by 15%. Furthermore, we found that the proportion of asymptomatic infection was as high as 13.7%. Assuming these events involving asymptomatic carriers are not isolated, perhaps more people will be infected in Lu'an. Therefore, we should be concerned about asymptomatic carriers, who represent an enormous challenge to containment in other countries responding to the COVID-19 epidemic.

Several limitations should be considered in interpreting our findings. First, since CISDCP is a passive surveillance system, mild cases or asymptomatic carriers who did not seek medical help may not have been identified. As a result, the number of COVID-19 cases may have been underestimated in our study. Second, the source of infection in at least five cases was not investigated clearly, and whether these cases were caused by an asymptomatic carrier or community transmission should be seriously considered.

## Conclusions

Our study showed that COVID-19 community transmission in Lu'an city was blocked effectively at the early stage, thus limiting the scale of the epidemic. Our results highlight the fact that asymptomatic spread may represent a substantial challenge in response to COVID-19. We believe it is beneficial to enhance the sensitivity of COVID-19 diagnostic tests and to increase the public health authority's capacity for surveillance.

## Data Availability

The data that support the findings of this study are available from the corresponding author upon request. Restrictions apply to the availability of these data, which were used under licence for this study.
